# Dual Bowel Obstruction: A Rare Case of Gallstone Ileus and Colonic Adenocarcinoma

**DOI:** 10.7759/cureus.21379

**Published:** 2022-01-18

**Authors:** Sarah Marie, Khalid A Alhejji, Sultanah Bin Gheshayan, Salah Bin Nafesah, Nahar Al Selaim

**Affiliations:** 1 Department of General Surgery, Ministry of National Guard-Health Affairs, Riyadh, SAU; 2 College of Medicine, King Saud bin Abdulaziz University for Health Sciences, Riyadh, SAU; 3 Medical Research, King Abdullah International Medical Research Center, Riyadh, SAU

**Keywords:** gallstone ileus, colonic adenocarcinoma, bowel obstruction, dual abdominal pathology, malignant colonic polyp

## Abstract

Bowel obstruction is a surgical emergency that leads to a high rate of admissions. Twenty percent of patients with acute abdominal pain will be diagnosed with bowel obstruction; eighty percent of them are of small origin. It is classified based on etiology to either mechanical or functional. Mechanical obstruction is a physical barrier that obstructs the passage of bowel content; it could be caused by adhesion, tumors, volvulus, hernias, strictures, and gallstone ileus. Functional obstruction is usually due to impaired peristalsis or metabolic disorders.

In this article, we report a case of an 80-year-old gentleman with no previous surgical history who was found to have a bowel obstruction. Diagnostic imaging and colonoscopy showed that his clinical presentation was due to gallstone ileus with cholecysto-enteric fistula and sigmoid mass. He underwent exploratory laparotomy with small bowel resection and sigmoidectomy with primary anastomosis and diverting ileostomy. The final pathology showed early moderately differentiated polyp adenocarcinoma T1N0 and was kept on surveillance.

The novelty of this case is the presentation of two different abdominal pathologies, which lead to large and small bowel obstruction. Thus, the management decision was challenging, and a thorough workup is advisable in such cases.

## Introduction

Bowel obstruction is a surgical emergency that leads to a high rate of admissions. Twenty percent of patients with acute abdominal pain will be diagnosed with bowel obstruction; eighty percent of them are of small origin [1]. It is considered a major cause of mortality, with a rate of 10 percent reaching 30 percent if left untreated or complicated by necrosis or perforation [2]. It can be furtherly classified based on etiology to either mechanical or functional [3].

Gallstone ileus is a complication of gallstone disease with a rate of 0.3 to 0.5 percent, which is common in elderly patients between 70 to 80 years of age. It represents one to four percent of overall causes of bowel obstruction [4]. It was described first in 1654 by Bartholin as a cause of mechanical obstruction due to the impaction of one or more large gallstones within the gastrointestinal tract. Its diagnosis is challenging, and in the majority of patients, it requires imaging in addition to clinical diagnosis. Rigler's Triad, which was described by Leo George Rigler in 1941, lists the elements needed for the diagnosis by imaging. It includes mechanical obstruction, pneumobilia, and an ectopic gallstone within the bowel lumen [5].

## Case presentation

An 80-year-old male with a past medical history of diabetes mellitus, and no prior history of surgeries, presented to the emergency department with a chief complaint of intermittent diffuse abdominal pain. The pain started suddenly, was colicky in nature, and lasted for three days. It was associated with constipation and vomiting. The patient recalled that a year earlier, he complained of abdominal pain, for which he sought medical attention and was diagnosed with cholelithiasis at another hospital.

Upon assessment, the patient was alert, oriented, showing signs of dehydration while maintaining his vitals with normal blood pressure and heart rate. His abdominal exam showed generalized distension with diffuse mild tenderness; there were no signs of peritonitis. Laboratory workup, including complete blood count, urea and electrolyte, liver, and coagulation profile, were unremarkable except for evidence of dehydration.

**Figure 1:**
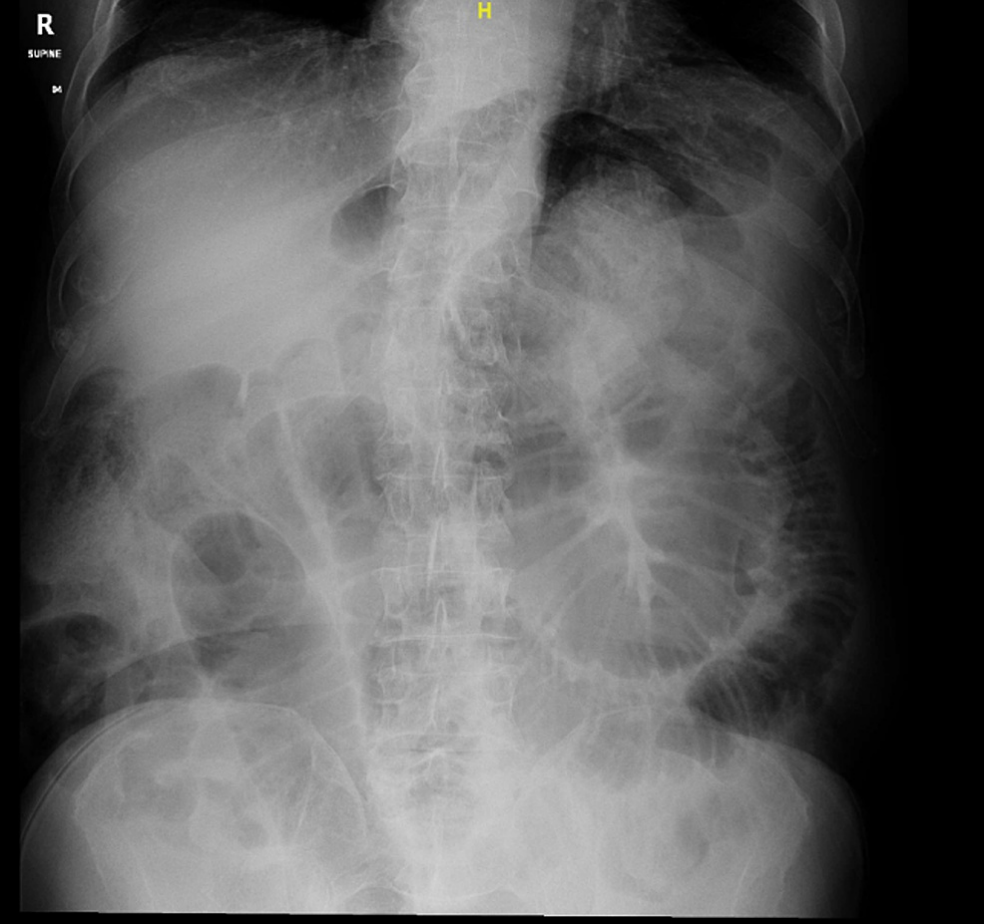
Abdomen X-ray showing dilated small bowel loops

Initially, the patient underwent abdominal radiography, which showed dilated small bowel loops without pneumoperitoneum (Figure 1).

**Figure 2:**
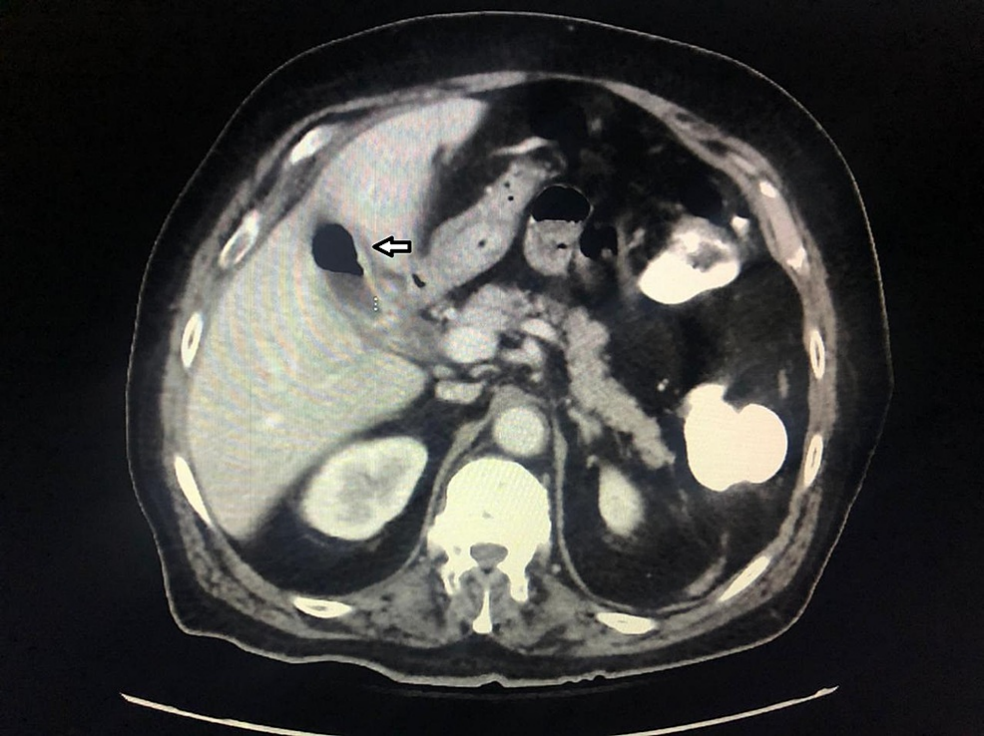
Abdomen CT with intravenous and oral contrast showing pneumobilia

Since the patient was clinically stable, he underwent an abdomen pelvis computed tomography (CT), which showed intermediate grade small bowel obstruction due to gallstone ileus with cholecysto-enteric fistula and the presence of pneumobilia (Figure 2).

**Figure 3:**
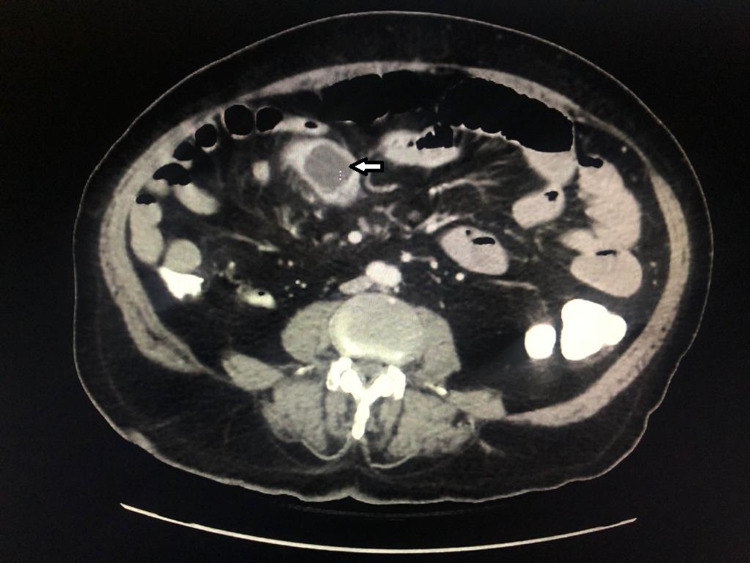
Abdomen CT with intravenous and oral contrast showing dilated bowel loops with a sigmoid mass

In addition, the CT showed sigmoid mass circumferentially obstructing the lumen (Figure 3). The patient was admitted for further workup and definitive management.

Resuscitative measures were taken: the patient was kept nil per oral, started on intravenous fluid, and a nasogastric tube was inserted. Since the patient was clinically stable, his abdomen exam was reassuring, and the oral contrast passed beyond the points of obstruction, he was further worked up. An urgent colonoscopy was performed, which showed fungating mass about 50 cm away from the anal verge, which was biopsied.

The scope passed to the terminal ilium with difficulty, and no other lesions were noted. Findings and the surgical options were discussed with the patient and family. He then underwent exploratory laparotomy with small bowel resection and sigmoidectomy with primary anastomosis and diverting ileostomy.

**Figure 4:**
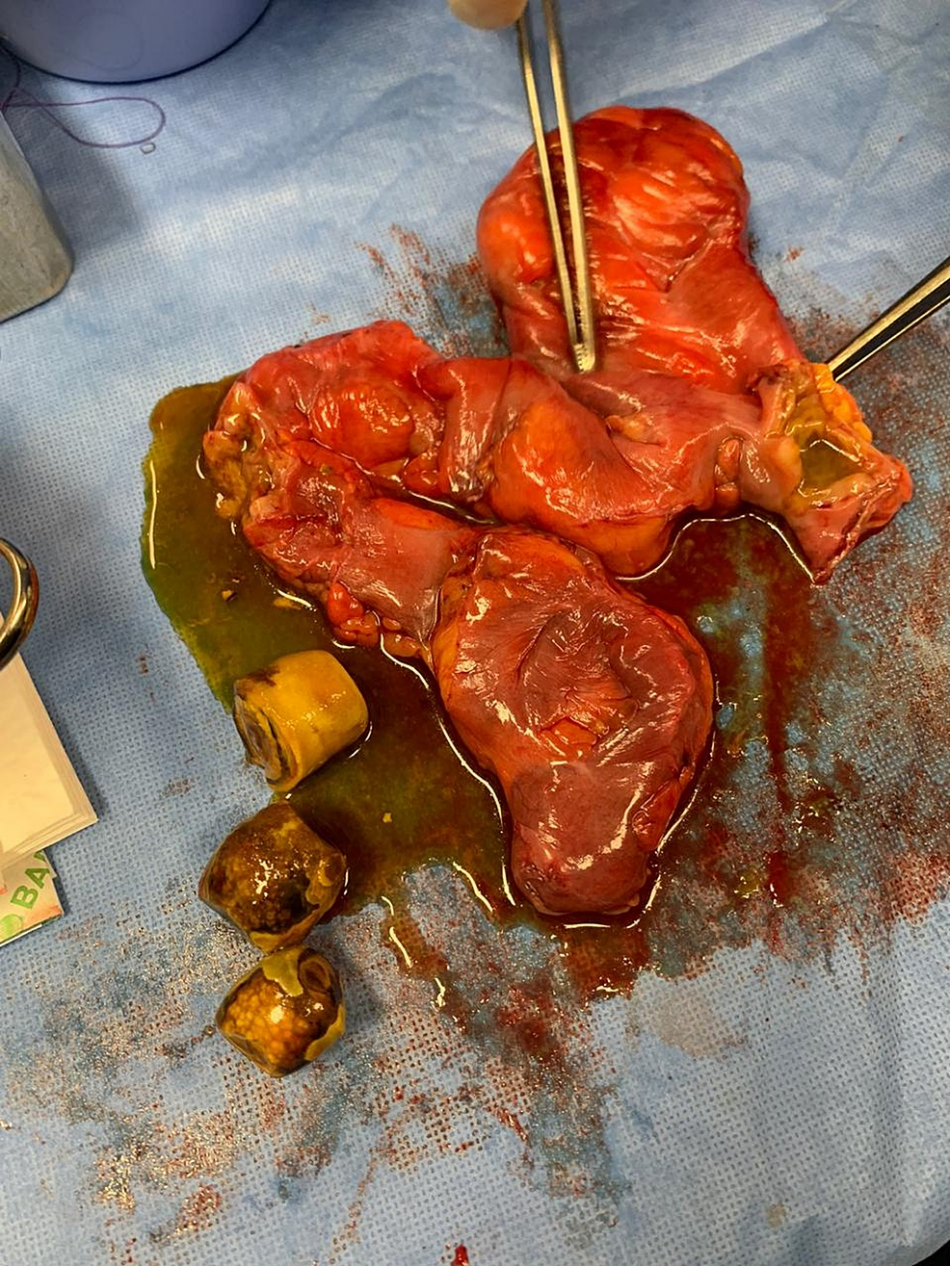
Bowel segment containing large gallbladder stones with fistula formation in-between

Intraoperatively, the gallbladder was not visualized as it was decided that a cholecystectomy be performed electively after the emergent procedure. The small bowel was mildly dilated, and around 120 cm from the ligament of Treitz, three stones were noted within the small bowel loops. These segments were adherent to each other with an area suspicious of fistula-like formation in between. This finding could explain the partial rather than complete obstruction he had. This segment was resected; both ends were utilized later for the creation of ileostomy and mucus fistula (Figure 4).

Then the attention was diverted to the sigmoid. A mass was noted proximal to the tattooed margin, which involved the whole lumen of the sigmoid. Oncological resection was obtained, and primary anastomosis was done. Given the nutritional status and frailty of the patient, a decision was made not to surgically manage the cholecysto-enteric fistula at the same time and to create a protective ileostomy.

**Figure 5:**
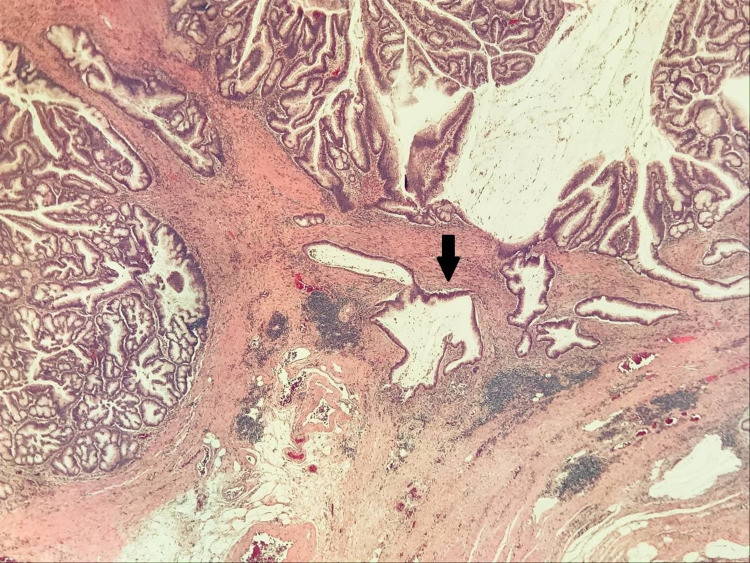
Histology slides showing adenocarcinoma of the colonic polyp

Final pathology showed early moderately differentiated adenocarcinoma T1N0M0 (Figure 5). The case was discussed in the tumor board, and it was decided that there would be no further management, and the patient would be under surveillance.

## Discussion

Large bowel obstruction accounts for 80% of total colorectal cancer-related emergencies. In 70% of the cases, the obstruction is distal to the splenic flexure. Despite its high prevalence, diagnosing and managing such cases remains challenging for surgeons. Different surgical options have been described in the literature to approach the obstruction. The management is customized according to the patient's clinical and functional status, tumor staging, and the presence of a specialized surgical team [6].

In our case, the patient presented with typical symptoms of bowel obstruction. Based on the patient's medical history, he was diagnosed with cholelithiasis years before his presentation to ER. In such cases, suspicion of gallstone ileus should always be of concern. Gallstone ileus is a rare pathological outcome of cholelithiasis; it is more common among elderly patients. However, gallstone ileus was reported by a group of colleagues in a 30-year-old gentleman [7]. Usually, it is caused by the presence of a single impacted stone ranging from two to 10 cm, with a mean size of 4 cm. As opposed to the common knowledge about this pathology, in this report, three gallstones were found intraoperatively within the patient's small bowel, which all were removed [8].

During the preoperative workup of the patient, the CT images confirmed the diagnosis and showed another pathology, which was the colonic polyp. Initially, it was thought that the presence of the polyp was an incidental finding, however with the role of colonoscopy, we managed to confirm that it was a partially obstructing mass. 

The usual treatment of gallstone ileus causing obstruction is the surgical removal through an enterolithotomy with possible segmental resection of the affected bowel. Primary anastomosis or ileostomy creation is then decided based on complication and viability of the tissue. In 2020, Nicholas et al. reported an unusual resolution of obstruction due to gallstone ileus by passing the stones with the bowel motion; however, conservative management was not proposed in the literature as the standard of care [9]. The literature supports that fistulotomy with cholecystectomy is performed in selected cases where patients are less co-morbid and surgically fit. This was the approach followed in the management of this patient [10]. The surgical team was able to resect the affected small bowel segment, and the creation of a protective loop ileostomy, in this case, was necessary because an anastomosis was done in the left colon. Up to date, resection and primary anastomosis are the preferable approach for left-sided colonic masses in the absence of complicated obstruction or perforation [11].

It is extremely rare to encounter multiple pathologies causing bowel obstruction on different bowel segments. Upon reviewing the literature, no cases reported similar presentations, which made the management decision more challenging. Involving a multidisciplinary team in the treatment plan was a crucial step in the management. 

## Conclusions

Bowel obstruction is a well-established abdomen disease with known etiologies. However, in special cases with elderly patients and multiple co-morbidities, a thorough assessment and a complete workup are indicated before a definitive intervention. 
